# Report: NIA workshop on translating genetic variants associated with longevity into drug targets

**DOI:** 10.1007/s11357-018-0046-7

**Published:** 2018-10-29

**Authors:** Nicholas J. Schork, Nalini Raghavachari, On behalf of Workshop Speakers and Participants

**Affiliations:** 1 Department of Quantitative Medicine, The Translational Genomics Research Institute, Phoenix, AZ USA; 2 National Institute on Aging, Bethesda, MD USA

**Keywords:** Health and life span, Integrative omics, Systems biology, Protective factors, Target identification

## Abstract

To date, candidate gene and genome-wide association studies (GWAS) have led to the discovery of longevity-associated variants (LAVs) in genes such as FOXO3A and APOE. Unfortunately, translating variants into drug targets is challenging for any trait, and longevity is no exception. Interdisciplinary and integrative multi-omics approaches are needed to understand how LAVs affect longevity-related phenotypes at the molecular physiologic level in order to leverage their discovery to identify new drug targets. The NIA convened a workshop in August 2017 on emerging and novel in silico (i.e., bioinformatics and computational) approaches to the translation of LAVs into drug targets. The goal of the workshop was to identify ways of enabling, enhancing, and facilitating interactions among researchers from different disciplines whose research considers either the identification of LAVs or the mechanistic or causal pathway(s) and protective factors they influence for discovering drug targets. Discussions among the workshop participants resulted in the identification of critical needs for enabling the translation of LAVs into drug targets in several areas. These included (1) the initiation and better use of cohorts with multi-omics profiling on the participants; (2) the generation of longitudinal information on multiple individuals; (3) the collection of data from non-human species (both long and short-lived) for comparative biology studies; (4) the refinement of computational tools for integrative analyses; (5) the development of novel computational and statistical inference techniques for assessing the potential of a drug target; (6) the identification of available drugs that could modulate a target in a way that could potentially provide protection against age-related diseases and/or enhance longevity; and (7) the development or enhancement of databases and repositories of relevant information, such as the Longevity Genomics website (https://www.longevitygenomics.org), to enhance and help motivate future interdisciplinary studies. Integrative approaches that examine the influence of LAVs on molecular physiologic phenotypes that might be amenable to pharmacological modulation are necessary for translating LAVs into drugs to enhance health and life span.

## Introduction

To date, candidate gene and genome-wide association studies (GWAS) have led to the discovery of longevity-associated variants (LAVs; mostly single nucleotide polymorphisms (SNPs)), such as those identified in the FOXO3A and APOE genes. Many of these LAVs have been replicated in multiple population cohorts and are thought to not only enhance longevity but potentially protect individuals from diseases as well (Flachsbart et al. 2017; Nebel et al. 2011; Willcox et al. 2006; Pilling et al. 2017; McDaid et al. 2017; Joshi et al. 2016; Sebastiani et al. 2017a; Partridge et al. 2018). As a result, it is believed that the identification of LAVs can lead to the identification of therapeutic targets to enhance outcomes, e.g., cardiovascular health, metabolic function, healthy aging, and ultimately life span. Unfortunately, translating a LAV to a drug target has been difficult at best. This can be attributed, in part, to (1) a lack of understanding of the molecular physiologic consequences of LAVs and (2) a lack of insight into how the genes affected by those LAVs interact with other genes in ways that might compromise direct therapeutic targeting of any one of those genes. This problem is not entirely unique to the study of LAVs, although the relatively low number of genome-wide significant SNPs associated with longevity compared with other traits (Partridge et al. 2018) does make it difficult to explore different strategies for translation. In addition, it may be the case that different aspects of aging are under the control of different genetically mediated processes as suggested by (Evert et al. 2003; Sebastiani et al. 2017b). Ultimately, gaining insight into the molecular physiologic consequences of LAVs to the point of understanding how one might modulate those consequences pharmacologically is not trivial, as the relationships among LAVs and gene expression, protein abundance and function, metabolite levels and other molecular factors, as well as broader human physiology, are complex. This suggests that there is a need for interdisciplinary, integrative (e.g., “multi-omics”) and “triangulation” (Munafo and Davey Smith 2018) approaches to understanding how LAVs impact longevity-related phenotypes if they are to reveal drug targets.

In order to expose the need for understanding how LAVs affect molecular physiologic factors that are amenable to pharmacologic manipulation, especially through the use of emerging data analytic and bioinformatics approaches, The National Institute on Aging (NIA) convened a workshop in August 2017 on emerging and novel approaches to overcoming current challenges in the translation of LAVs to drug targets (NIA workshop on “Integrative Omics Approaches to Discover Influential Mechanistic Pathway(s)/Molecules Associated with LAVs for Healthy Aging”). The goal of the workshop was to identify ways of enabling, enhancing and facilitating interactions among researchers from different disciplines focusing on the identification of LAVs and the mechanistic/causal pathway(s) and protective factors they influence for drug targeting purposes. The workshop included participants with expertise from fields such as biogerontology, bioinformatics and biostatistics, systems biology, translational genomics, comparative biology, epidemiology, and human genetics and led to discussions surrounding future integrated research directions that could potentially identify targets and/or therapeutics for healthy aging.

The discussions among the workshop participants resulted in the identification of critical needs in several areas, as well as potential strategies for addressing them. These included (1) the initiation and better use of cohorts with multi-omics profiling on the participants, (2) the generation of relevant longitudinal information on multiple individuals, (3) the collection of data from non-human species (both long and short-lived) for comparative biology studies, (4) the refinement of computational tools for integrative analyses, (5) the development of novel computational and statistical inference techniques for both assessing the potential of a drug target and determining if any available drugs could modulate that target in a way that could provide protection against age-related diseases and/or enhance longevity, and (6) the development or enhancement of databases and repositories of relevant information, such as Longevity Genomics website (https://www.longevitygenomics.org), to facilitate and motivate relevant interdisciplinary studies. The ultimate consensus conclusion of the workshop participants was that integrative approaches to the exploration of the influence of LAVs on molecular physiologic phenotypes that might be amenable to pharmacological modulation are indeed necessary for translating LAVs into longevity and health-enhancing drugs. In the following, we provide a discussion of additional motivating factors for the workshop, the intellectual and logistical organizing principles behind the workshop, summaries and highlights of the individual presentations, and specific suggestions emerging from the workshop, including suggestions about activities that were not explicitly represented at the workshop.

## Aging as a complex process

Aging, manifested by a gradual decline of normal physiological functions over time, is a complex multifactorial process mediated by highly intertwined biological mechanisms (Sierra and Kohanski 2017) that increase susceptibility to many diseases, including cancer, metabolic disorders, cardiovascular disorders, rheumatic, bone-related, and neurodegenerative diseases such as Alzheimer’s disease (AD) (Brunet and Rando 2007; Brunet and Sedwick 2015; Kaeberlein et al. 2015; Kennedy 2008). There are, however, individuals who survive well beyond average life expectancy and spend a short period of their lives with age-related diseases or disability (Perls 2002; Perls et al. 2000). These individuals often remain largely free from general age-related morbidities. Epidemiological and population-based studies across the world focusing on these exceptionally long-lived individuals have provided insight into the factors, and interactions among them, which appear to be associated with the reduced morbidity, reduced mortality, and survival to older ages that they exhibit (Willcox et al. 2006; Perls 2002; Perls et al. 2000; Newman and Murabito 2013). Further, genetic studies of exceptionally long-lived individuals suggest that they may possess genetically mediated factors that modulate fundamental biological processes underlying aging and protect them from developing many age-related diseases (Andersen et al. 2005; Barzilai et al. 2012; Milman and Barzilai 2015). Genetic studies of exceptionally long-lived individuals are also valuable for identifying protective factors and potential therapeutic targets that promote healthy aging, by either protecting individuals from developing age-related diseases, modulating fundamental mechanisms of aging in a positive way, or both. Further, it is believed that slowing the rate of aging has the potential to protect against the development of multiple conditions instead of preventing any single disease (Sierra and Kohanski 2017; Kaeberlein et al. 2015; Kennedy and Partridge 2017; Kennedy and Pennypacker 2014). Based on this premise, the NIA has supported research seeking to identify genetic variants (both common and rare) associated with healthy aging and longevity through, e.g., the funding of GWAS and sequencing studies focusing on exceptionally long-lived individuals. Relevant GWAS and sequencing studies pursued to date have suggested that identifying genome-wide significant LAVs is not trivial and, in fact, only variants in the ApoE and FOXO3A genes have been consistently replicated as associated with longevity across different cohorts (Partridge et al. 2018). In addition, complementary hypothesis-driven candidate gene studies involving model organisms have identified several genes associated with longevity or decreased mortality rates (Yanai et al. 2017). These findings have motivated, or could motivate, testing variants in the human orthologs of those genes with longevity in human association studies. In fact, variants considered in human association studies that have been motivated in part by studies of the orthologs of the genes harboring those variants in model organisms have implicated variants in or near many lipoprotein metabolism genes and include the APOE, FOXO, insulin/IGF1 signaling pathway genes, LMNA, RNA editing genes, and HSF227 genes (Nebel et al. 2011; Willcox et al. 2006; Conneely et al. 2012; Morley and Morimoto 2004; Sebastiani et al. 2009). This suggests that model organism studies, as well as studies that focus on age-related diseases and factors, like lipoproteins, can inform studies of human longevity and the identification and validation of LAVs. It should be noted, however, that many genes shown to influence lifespan in model organisms have human orthologs that have not been shown to either influence human longevity or harbor variants associated with human longevity-related phenotypes. Although there are strategies for studying these genes (e.g., determining if other genes in pathways and processes involving the genes in question do harbor variants associated with human longevity), many genes found to be associated with lifespan in model organism studies simply do not have good and reliably determined orthologs in humans. Such genes were not the focus of the NIA workshop, so they will not be addressed here.

## Translating LAVS into drug targets

The translation of genetic variants associated with a particular disease or phenotype, such as LAVs, into viable and compelling insights into drug targets has been slower than expected. In fact, drugs based on insights arising from studies of the effects of LAVs in the APOE gene—a very compelling gene biologically and one that harbors variants whose associations with human phenotypes have been replicated more than any other set of variants—have been largely unsuccessful. This is evidenced by failures in phase III clinical trials of drugs designed to treat Alzheimer’s disease (AD) that target genes in the APOE gene pathway (Amanatkar et al. 2017; Sperling et al. 2011; Sperling et al. 2013). This slow progress can be attributed to at least three factors. First, there are currently no efficient, high-throughput and reliable ways of obtaining fundamental functional information on linear, causal genotype-phenotype relationships (if any) that could reveal potential drug targets mediated by variants identified from genetic association studies, although some progress in this area is being made (Tewhey et al. 2018; Tewhey et al. 2016). Second, there is a general lack of understanding of the mechanistic action of LAVs that considers pleiotropic, non-linear, and epistatic influences affecting genes that might induce feedback and redundant processes that thwart easy understanding of the molecular physiologic effects of individual LAVs. Third, there is no consensus on how one might be able to leverage model species to understand the influence of LAVs on the nuanced and potentially unique physiology of humans that facilitate and ease the identification of human drug targets.

**Fig. 1:**
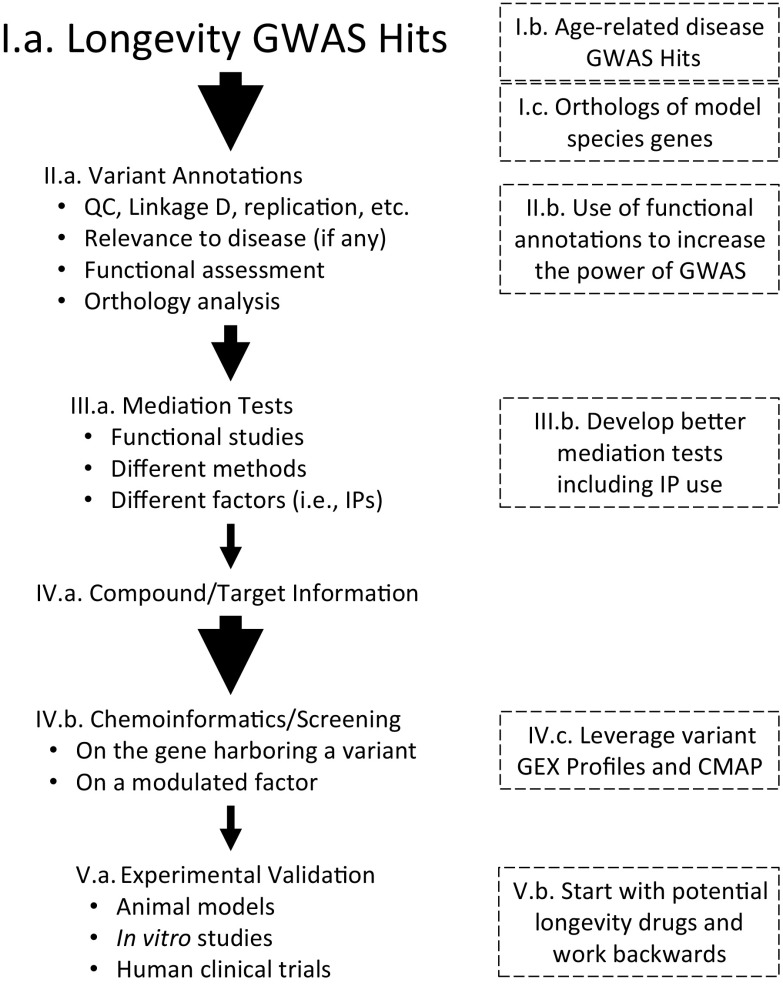
Graphical depiction of “filters” used in the flow of information and analyses for taking information about genetic variants associated with human lifespan (LAVs) and identifying potential compounds that might modulate factors mediated by those genetic variants (see text). The size of the arrows corresponds to the relative number of factors considered at a point in the workflow. Dashed boxes indicate areas where extensions or additional analyses and resources might be exploited to enhance the information flow based on suggestions by the workshop participants

One way to conceptualize the issues associated with the translation of human LAVs into drug targets is to consider the flow of information from the discovery of a LAV to the validation of a drug that targets a factor mediated by that LAV. Figure 1 depicts this translation information flow by positing “funnels” and “filters” used to differentiate questionable genetically mediated targets from more biologically sound genetically mediated targets, as well as less compelling candidate compounds to modulate a target from more compelling candidate compounds to modulate a target. Thus, the first step (“I.a” in Fig. 1) involves identifying LAVs. Increasing the pool of LAVs will obviously lead to more potential drug targets. The second step (II.a) involves characterizing (or “annotating”) the gene or functional element the LAV resides in. This is not always trivial. The third step (III.a) involves identifying a factor whose activity is mediated by the LAV that can potentially be modulated pharmacologically (e.g., the expression level of a gene, the abundance of a protein or metabolite). This is often pursued with in vitro and model organism studies but can be pursued with special statistical analysis methods that use unique database resources known as “mediation” analyses, as discussed later. The fourth step (IV.a) involves identifying a source of well-annotated potential compounds whose effects on a target can be evaluated. This step can often be pursued through special computational techniques and databases that often fall under the heading of “chemoinformatics” (IV. b). The final step (V.a) involves testing a candidate compound for in vivo effects on longevity and longevity-related phenotypes.

The workshop participants discussed research efforts that reflect different components of the information flow depicted in Fig. 1, with an emphasis on items 1–3, and a goal of seeing how their work could be complementary to others’ work and lead to more efficient translation of LAVs into testable drug targets. The participants had a very diverse set of scientific backgrounds and expertise that included biogerontology, bioinformatics and biostatistics, systems biology, translational genomics, comparative biology, epidemiology, and human genetics. The discussions that the participants engaged in led to potential insights into ways of enhancing the translation of LAVs into drug targets (noted by the boxed items in Fig. 1, which will be referenced in remarks about the presentations below) as well as proposals for the exchange and creation of resources that could expedite this translation.

## Workshop organization

Given the complexities surrounding, and multidisciplinary efforts needed for, the translation of LAVs into drug targets, questions about how best to integrate different sources of information and expose gaps in current translational paradigms arise. For example, one could ask if greater emphasis on assessing and critiquing the longevity-related phenotypes used in genetic association studies makes sense given the various definitions researchers have developed, or if generating proteomics, metabolomics, and other omics data, perhaps collected longitudinally, should be a priority since this may yield a greater number of potential drug targets associated with LAVs. Alternatively, should greater emphasis be given to the development of experimental strategies, including those involving model species, to validate computational models linking LAVs to molecular biological processes amenable to pharmacological modulation? Or should greater emphasis be given to actual intervention studies focusing on a potential mechanism associated with a LAV? What about greater emphasis being placed on developing more comprehensive, integrated, and queryable databases harboring relevant information about LAVs, their scientific support, and their likely influence on modifiable molecular physiologic processes? Or should greater emphasis be placed on statistical and computational methods for drawing compelling inferences from integrated data sources?

In the light of these and related questions, the workshop was designed as a series of brainstorming sessions involving a group of experts to evaluate how different approaches to identifying and validating longevity-enhancing drug targets based on LAVs can be brought together and integrated. The workshop was divided into four different sessions: “omics analytical approaches,” “population studies,” “comparative biology,” and “ success stories and challenges,” each addressing current challenges in the identification of putative mechanistic/causal pathways affecting longevity based on the identification of LAVs. Each participant was asked to consider ways in which future studies of LAVs would bridge genetics, genomics, proteomics, metabolomics, connectomics (i.e., network biology), and comparative biology. In addition, the participants were asked to consider how these efforts could involve collaborations between private-public organizations and the development of appropriate resources (data and/or biospecimen repositories) that might be necessary. Summaries of the comments by the individual participants and the ensuing discussions are provided below in the order in which speakers gave their presentations. We reference Fig. 1 where appropriate to provide context and place suggestions for improvements within the proposed translational workflow.

## Workshop summaries

### Workshop introductory remarks

Nalini Raghavachari and Evan Hadley from the NIA introduced the workshop with some brief remarks about its motivation and logistics. Nicholas Schork, who along with Drs. Raghavachari, Hadley, Chhanda Dutta, Max Guo and Suzana Petanceska, organized the workshop, then provided an overview of the challenges of translating LAVs to drug targets, including a few basic strategies that led to drugs designed to treat age-related conditions using genetic information (Chen et al. 2008; Bulawa et al. 2012). He also discussed the challenges of developing computational infrastructure for integrating large amounts of omics data, especially leveraging insights from studies of model species, and challenged the workshop participants to think “outside the box” about what they may wish they had in their possession to facilitate translational studies involving LAVs.

### Session 1: omics analytical approaches

Nicholas Schork continued his introductory remarks but focused on the development of statistical analysis methods for conducting Mendelian randomization (MR) tests (Davey Smith and Hemani 2014), which can assess the likely causal relationship between a LAV; an intermediate phenotype (IP) amenable to pharmacological modulation, such as gene expression levels; and a longevity-related phenotype, such as life span (see item III.a in Fig. 1). Some MR tests use imputation strategies for assigning individuals with genotype data IP values based on known associations between genetic variants and those IPs. These imputed or assigned IPs can then be tested for association with a longevity-related phenotype in lieu of having them directly measured. MR tests thus have great utility because of the ease with which they can be conducted but are only as reliable as the connection between the IP and the genetic variants used for imputing them. In addition, the development of more powerful MR statistical methods and the development of resources and databases harboring information about genetically mediated IPs would benefit the translation of LAVs into drug targets (item III.b in Fig. 1). Dr. Schork also discussed bioinformatics approaches for “systems level orthology” assessments. These assessments consider similarities between genetically mediated phenomena in a model species and genetically mediated phenomena in humans in order to make claims about why there may be inconsistencies in the results of studies involving a model species and the results of a study of humans. Such efforts could enhance the number of candidate genes to be considered in human genetic association studies (item I.c in Fig. 1) and also help validate potential LAV-mediated drug targets (item III.b in Fig. 1). Finally, Dr. Schork mentioned ongoing activity to develop the “longevity genomics” web resource harboring information about strategies for conducting MR tests. This web resource could be used to integrate additional information of interest to translational researchers working on human longevity (https://www.longevitygenomics.org).

Goncalo Abecasis summarized the “Trans-Omics for Precision Medicine (TOPMED; https://www.nhlbi.nih.gov/science/trans-omics-precision-medicine-topmed-program)” initiative. TOPMED is exploiting rapid advances in genome sequencing and genotyping technology to enable very large population and clinical studies of human genetic variation. This initiative could lead to, among other things, novel LAVs (item I.a in Fig. 1). TOPMED plans to analyze > 50,000 deeply sequenced human genomes, corresponding to thousands of billions of bases of raw sequence data. The generation, transfer, and analysis of the data present many opportunities for scientific discovery. This includes the identification of LAVs, but also, importantly, genetic variants contributing to age-related diseases that may overlap with LAVs (item I.b in Fig. 1). TOPMED will require addressing a number of computational and analytical challenges given its scope and size, and also likely expose new opportunities to develop and implement analytical strategies for identification of genetic associations (item III.b in Fig. 1), as well as modes of data sharing.

Gibran Hemani discussed an initiative in the UK to develop “MR-Base,” a platform for conducting MR tests that exploits access to summary data from some of the largest GWAS datasets in the world (Davey Smith and Hemani 2014; Zheng et al. 2017). MR-Base (https://www.mrbase.org) provides the user with choices about the genetic variants they want to consider in an MR test, as well as a variety of IPs and phenotypes to choose from and is therefore of great utility in the pursuit of purely computational SNP-based mediation tests (items III.a and III.b in Fig. 1). MR-Base also provides the user with access to a variety of online statistical methods to conduct MR analyses, so there is no need for porting or harmonizing data to conduct certain analyses. MR-Base includes data from studies on longevity and hence can be used to interrogate potential causal pathways leading from a LAV to longevity-related phenotypes. The workshop attendees discussed the idea that MR-Base could be leveraged, if not simply referenced with an appropriate link to its website, in online resources such as the Longevity Genomics website (https://www.longevitygenomics.org).

**Fig. 2:**
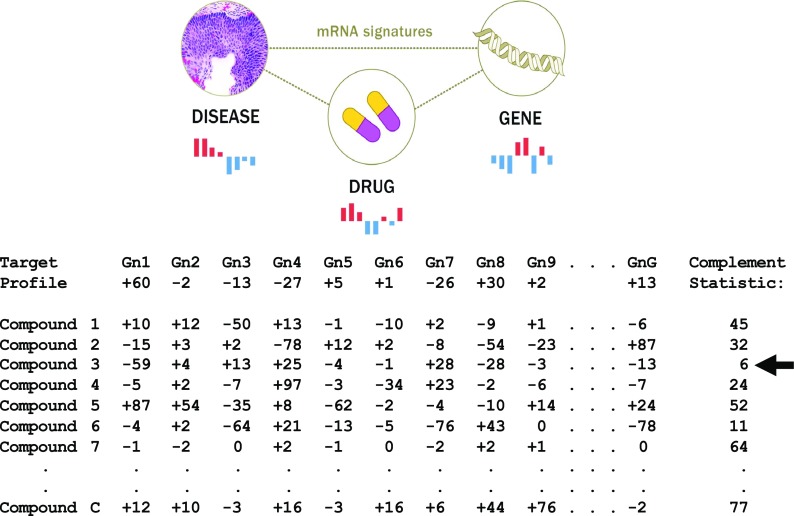
Basic depiction of the principle behind the “connectivity map (CMap).” Upper panel: gene expression fingerprint connections exploited by the CMap. Lower panel: Hypothetical use of the CMap. Gene expression profile over G genes (“Gn1, Gn2,…, GnG”) of a “target” (say from the diseased tissues of a specific patient or based on averages over many patient profiles) is obtained. Some genes in this profile are overexpressed (e.g., Gn1 by 60 units) and some are under expressed (e.g., Gn4 by − 24 units) relative to some standard. C different compounds have been previously assessed for how they change the expression levels of genes, ideally in the same tissue type as harvested from the target. A metric is used to identify compounds that affect the gene expression levels in the opposite manner to those observed in the patient profile using a statistic, such as summing up the differences between the compound effects and the target profile on gene expression (e.g., the “complement statistic”). The compound which appears best at reversing the pathogenic gene expression signature (i.e., exhibits a complement statistic closest to 0.0 and seemingly has effects which cancel out the over- and under-expressed genes in the target profile; in this instance the compound identified with the black arrow) is seen as good candidate for treating the condition associated with the target profile

Ted Natoli provided an update on the Connectivity Map (CMap), which is a public domain tool for determining if a gene expression “fingerprint” associated with a particular condition (e.g., tumors in a cancer setting; referred to as a “query condition”) is known to be consistent with the gene expression profile induced by certain drugs (Subramanian et al. 2017; Lamb 2007; Lamb et al. 2006). Figure 2 depicts some of the essential ideas behind the CMap. The CMap can be used to identify drugs and natural products that may be worth investigating for the query condition of interest. The CMap has its limitations, but the amount of data it draws on to accommodate connections between drug effects and gene expression fingerprints is being expanded. In the context of the translation of LAVs into drug targets, a discussion occurred among the workshop participants about how researchers might be able to identify gene expression fingerprints in cells or tissues harboring a LAV, which could then be assessed with the CMAP to identify drug candidates of relevance to aging (items IV.b and IV.c in Fig. 1). Gene expression profiles, and lists of drugs that could generate them, based on a CMAP analysis of cells and tissues harboring a LAV could also be used to annotate the relevant variants if the effects of those variants are unknown (item II.a in Fig. 1).

**Fig. 3:**
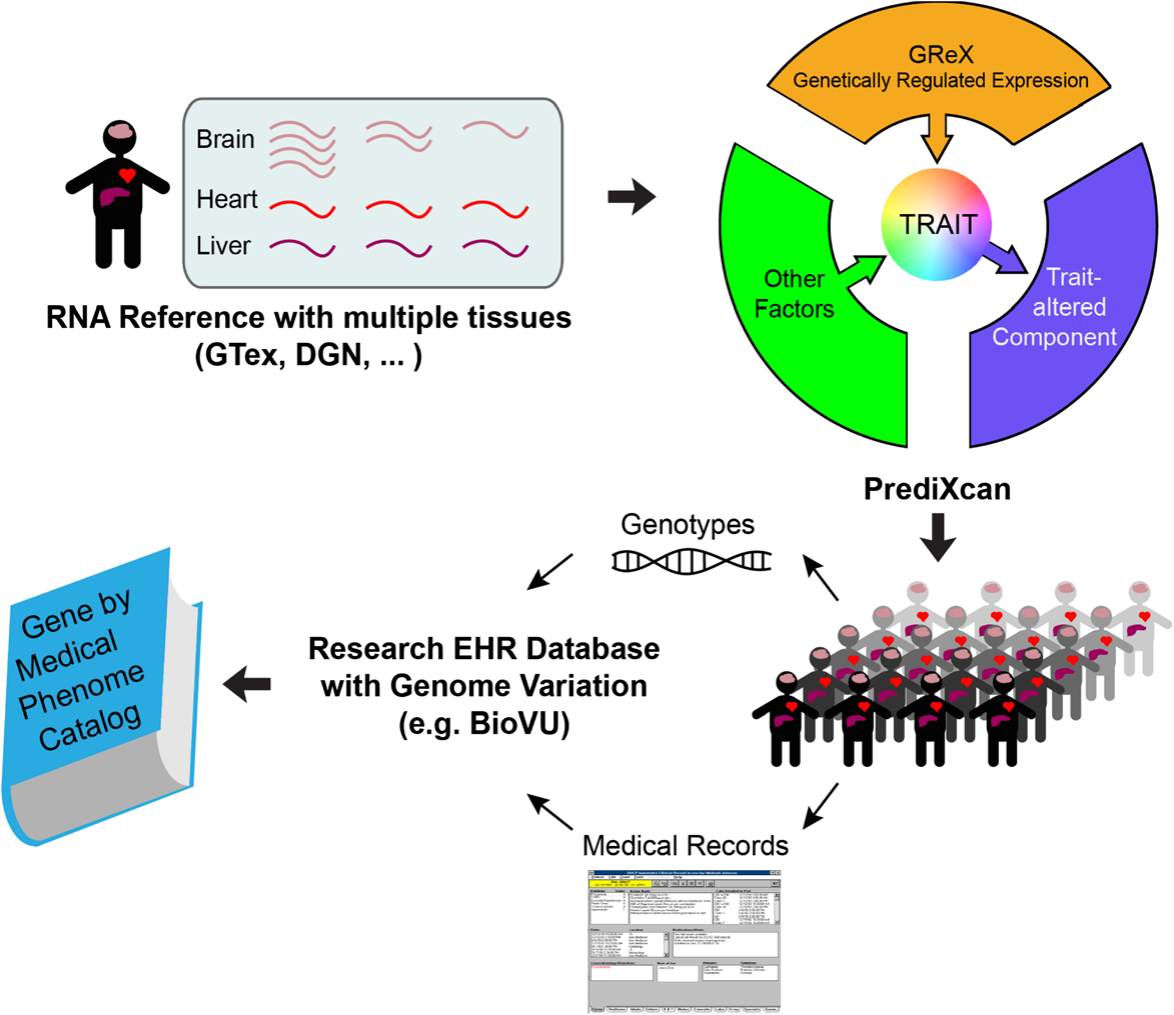
Overview of the components of the “BIoVU” catalog at Vanderbilt University and how it is being used to identify potential factors mediating diseases that might be amenable to pharmacologic modulation. The catalog contains health records on over 240,000 people (2.6 million records total), 40,000 with genomic variant data. Genotype and other data from GTex and other resources are used to impute tissue-specific gene expression levels to individuals in the BIoVu database, and these imputed expression levels are assessed for correlations with different disease traits

Nancy Cox detailed the infrastructure associated with, and her experience using, the “BioVu” database at Vanderbilt (https://victr.vanderbilt.edu/pub/biovu/). BioVu is a very large database harboring clinical information and electronic medical record-derived data on patients in the Vanderbilt health system who have also been genotyped. The combined genotype and phenotype information allow researchers to investigate associations between genetic variants and clinically relevant phenotypes. This includes age-related diseases (Sierra and Kohanski 2017) as well as a number of IPs that have been collected on the patients, such as clinical chemistries, inflammatory markers, etc. (items I.a and I.b in Fig. 1). Dr. Cox also described her team’s efforts to develop novel analysis methods for conducting MR tests and using the BioVu data as a platform for vetting and applying them (Fig. 3).

### Session 2: population studies

**Fig. 4:**
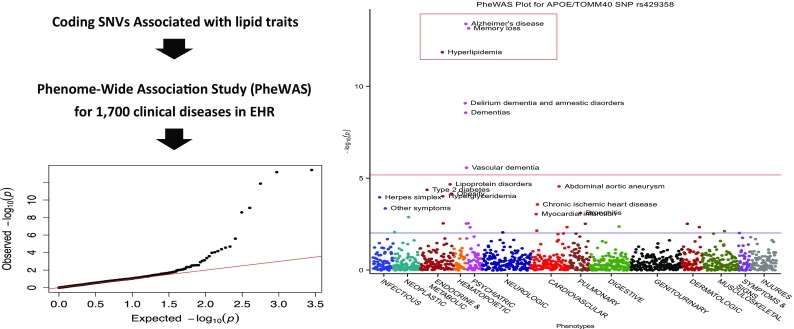
Graphical depiction of a proof-of-principle “phenome-wide association study” (PheWAS) pursued by Regeneron and Geisinger. Coding SNVs were found to be associated with lipid-related traits. One of the lipid-associated variants lied in ApoE/TOMM40. This variant was tested for association with 1 = 1700 phenotypes in the Geisinger electronic health record (eHR) database (left panel, which includes a q:q plot showing that a number of associated variants (black dots) exhibited such a pronounced association that they deviate from expectation (red line) and hence are not likely statistical false-positive associations). Variants in the ApoE/TOMM40 region exhibited the strongest associations with phenotypes in Geisinger’s electronic health record and phenotype database, including AD diagnosis, memory loss, and hyperlipidemia (right panel, red boxed items, in the plot depicting the strength of the association (y-axis) against phenotypes extracted from deidentified eHR data)

Alan Shuldiner presented aspects of ongoing activities at the Regeneron Genetics Center, which is collaborating with the Geisinger Health System to create a large-scale gene-phenotype database composed of hundreds of thousands of patients in the Geisinger Health System that have undergone exome sequencing (https://www.regeneron.com) (Dewey et al. 2016). By linking genetic variation with disease-related phenotypes, Regeneron and its collaborators hope to identify novel drug targets. Dr. Shuldiner emphasized that the current approaches to identifying drug targets based on pre-clinical models (e.g., purely computational, in vitro or model organism-based models) do not often result in drugs that exhibit appropriate activity in humans. He provided an example of how leveraging a large number of phenotypes that are tested for association with genetic variants in the GWAS setting (known as “phenome-wide association studies” or “PheWAS”) can aide in the discovery of drug targets, even with cross-sectional data alone (i.e., no longitudinal data; items I.a and I.b in Fig. 1 as well as items III.a and III.b in Fig. 1). Shuldiner and colleagues conducted a proof-of-principle study (Fig. 4) involving variants in the ApoE/TOMM40 gene region and associating them with 1700 phenotypes thought to contribute to 9 different diseases. They found that the strongest associations involved a SNP previously implicated in Alzheimer’s disease (AD). This SNP was found to be associated with dementia and hyperlipidemia in their database, suggesting that testing multiple phenotypes for association with a single variant or small subset of variants could aid in the identification, validation, and broad potential clinical relevance of LAVs as drug targets.

Steve Cummings described an approach to uncovering LAVs that involved associating genetic variants with a phenotype made up of a health/biomarker profile that indicates whether or not an individual is healthier than one would expect given that individual’s chronological age (items I.a and I.b of Fig. 1) (Kaeberlein 2018; Kim and Jazwinski 2015). The construction of this measure involves aggregating and scoring traditional disease risk factors to determine an individual’s likely overall disease risk as well as how many diseases they manifest. A measure was obtained from individuals within a number of cohorts associated with the NIA-funded Longevity Consortium (https://wp.longevityconsortium.org). Dr. Cummings applied this measure to individuals in four different cohorts that had a total of over 25,000 people at least 65 years of age with a number of measurements made on them related to aging. Multivariate statistical models that related the biological aging score and its components to survival endpoints were employed, although the analyses presented some challenges due to missing data and a need for harmonization of the phenotypes. These analyses suggested that walking speed, red blood cell distribution width, insulin sensitivity, height, and weight loss are good predictors of survival. Dr. Cummings also pointed out some of the challenges and need for removing barriers in sharing data and making legal agreements between industry and academic collaborators that arose during his studies.

Sudha Seshadri described the rich collection of resources, including both omics data and stored biospecimens, which includes 40,000 collective phenotypes from ~ 5000 participants (e.g., serum, plasma, RNA, DNA, induced pluripotent stem cells (iPSCs), and brain tissue) in the Framingham study (https://www.framinghamheartstudy.org) (Mahmood et al. 2014) The Framingham study is one of the oldest longitudinal studies on record and includes three generations of participants that could potentially be used for data-mining for aging studies in future. Many of the participants have been genotyped, enabling the discovery of potential LAVs (items I.a and I.b in Fig. 1). In addition, the variety of phenotypes collected on the participants in the Framingham study, many of which have been collected longitudinally, also provide a platform for implicating unique IPs that might be influenced by LAVs (item III.b in Fig. 1).

Joanne Murabito provided details on the resources that are available from the CHARGE consortium that could be leveraged for discovering LAVs (items I.a and I.b in Fig. 1) and embarking on integrated analyses to validate drug targets for healthy aging. The CHARGE consortium (www.chargeconsortium.com) is made up of 10 international cohort studies and more than 80 total collaborating studies for which some measures are in common including individual genotype data. The consortium collectively has a great deal of data, including, in some instances, omics data obtained from longitudinal cohort studies that could be a very rich source of IPs for mediation studies (items III.a and III.b in Fig. 1). Dr. Murabito also emphasized the need for computational approaches to harmonize phenotypic measures and data to enable combined or joint analyses across the CHARGE consortium cohorts. Summary results from some CHARGE phenotype GWAS are available in dbGaP and GRASP databases (https://grasp.nhlbi.nih.gov/Overview.aspx). It was suggested that web-based data aggregation and dissemination resources such as the Longevity Consortium and Longevity genomics websites (https://www.longevitygenomics.org) could be good vehicles for integrating non-proprietary information arising from the efforts of the CHARGE and related consortia.

Martin Hofmann-Apitius provided a discussion on the European Union (EU) efforts surrounding a public-private partnership on translational, pre-competitive research, known as the “Innovative Medicines Initiative” or “IMI.” One focus of the IMI is Alzheimer’s disease (AD) (Hofmann-Apitius et al. 2015), with the three projects: “AETIONOMY,” “EPAD,” and “EMIF-AD” as the initial projects. In addition, Dr. Hofmann-Apitius also provided some insights on a related initiative that focuses on informatics and data sharing: the “European Medical Information Framework (EMIF)” Initiative and its data catalog which includes all major clinical studies in the area of AD, and in addition provides access to millions of data points obtained from electronic health records (EHR) of patients in different health systems that can be re-used for LAV and IP discovery and related activities (items I.a and I.b as well as items III.a and III.b in Fig. 1). Dr. Hofmann-Apitius emphasized the importance of data sharing, which still remains a major obstacle in current research. He mentioned that well-organized collaborations, so-called mirror studies, across the world could assist in systematically exploring and verifying findings from major studies. He also suggested new strategies for leveraging clinical data in a way that overcomes ethical problems and public concerns on patient data privacy, a strategy based on virtualization of real-world clinical studies. The resulting “virtual cohorts” could be put in place, essentially promoting and maintaining the value of patient-derived information for translational research whilst eliminating the risk of compromising patient data privacy. He elaborated on the generation of virtual cohorts and their usage in data-mining and knowledge integration. It was noted by the workshop participants that given the complexities surrounding amassing, analyzing, and disseminating information about genes and drugs from population-based, model organism, and in vitro studies, exposing the experiences and resources like those associated with the IMI and EMI could be invaluable for the translational genomics community interested in LAVs and drugs. This is particularly true if one considers the fact that it might be possible to start with a drug thought to influence longevity or an age-related disease (such as metformin or rapamycin) and then interrogate the gene targets of that drug for variants that might be associated with longevity (i.e., and hence essentially be recognized as a bona fide LAV) or an age-related disease. Working backwards in this manner to the flow of information in Fig. 1 could be just as revealing about the role of genetic variation in longevity drug targeting as working forwards (item V.b in Fig. 1).

### Session 3: comparative biology

Caleb Finch opened the Comparative Biology session by considering the often neglected phenomenon of gene-by-environment interactions and how recent investigations, including his own, have shown how important they can be in studies of health span and longevity (Finch 2010). Dr. Finch presented data suggesting that tobacco smoke can induce and exacerbate AD pathobiology. He further suggested that since the global human exposure to toxic carbonaceous particles from tobacco or fossil fuels is very recent (probably within the last 5–10 generations) AD could be a modern disease arising from the introduction of evolutionarily novel environmental stressors in the form of these toxins. “Modulation” in the form of the reduction of these toxins could therefore reduce age-related cognitive decline and AD incidence. Thinking about the role of environmental factors in mediating a variant effect on a phenotype could be crucial for identifying LAVs, since it could reveal important contexts in which GWAS are pursued (item II.b in Fig. 1).

Richard Miller discussed a wide range of studies in his lab that focus on genes that contribute to longevity in a diverse set of species, including birds and mice (Miller et al. 2011). Dr. Miller has observed evidence that similar genetically mediated processes appear to be contributing to longevity in independent clades within the tree of life, potentially revealing a set of ubiquitous aging and longevity-related pathways that might be amenable to pharmacological modulation. The human orthologs of these genes could be studied in human association studies (items II.a and II.b in Fig. 1). Dr. Miller also discussed his experience as a contributor to the “interventions testing program (ITP),” which is funded through the NIA to test candidate longevity-enhancing compounds in mice (Nadon et al. 2017). The drugs tested in the ITP could involve those that target IPs revealed from studies of LAVs (item IV.b in Fig. 1). A handful of compounds tested have been shown to extend longevity, pointing to specific gene targets as starting points for human genetic association studies (item V.b in Fig. 1). One such compound arising from the ITP, rapamycin, is currently being tested for its effects on health span in companion dogs in randomized clinical trials (Urfer et al. 2017).

Steve Austad described his experience exploring the use of proteomics technologies in studying factors that contribute to longevity in a wide variety of species, including extremely long-lived bivalve mollusks (e.g., clams, scallops and oysters), which can live more than 500 years (Gruber et al. 2015). The proteins encoded by genes in model species that have reliable orthologs in humans could be examined in human studies and provide a very rich source of potential IPs for functional studies of genes harboring LAVs (item III.a and III.b in Fig. 1). In addition, the use of genes that are orthologous to those in model species that affect longevity could lead to better functional annotations for use in prioritizing variants in GWAS (item II.b in Fig. 1). In addition, studying proteins associated with longevity could help validate studies implicating the genes (for example those harboring LAVs) that encode those proteins.

Daniel Promislow provided a detailed summary of his studies exploiting metabolomics to identify longevity-associated metabolites in a wide variety of species (Hoffman et al. 2017). Unlike genes, transcripts, and proteins, which differ across species because of evolutionary-mediated changes in the DNA, RNA, and amino acid sequences, respectively, individual metabolites have unique chemical signatures whose abundances result, in part, from genetically mediated processes. Metabolites can hence be identified in any species that harbors those metabolites in the same way without having to deal with issues surrounding evolutionarily mediated sequence differences and orthology. Studies of metabolite profiles in long-lived individuals or species can identify favorable changes in metabolism (Mastrangelo and Barbas 2017). Individual metabolites reflecting those changes could then be potential targets for pharmacologic modulation, or be considered potential pharmacological agents themselves. In this light, metabolite levels can be used as ideal IPs in MR studies as well as the focus of functional studies (items III.a and III.b in Fig. 1).

### Session 4: integrative analysis: success and challenges

Ed Liu discussed issues surrounding the identification of gene-phenotype relationships given the vast amounts of genetic data that have been generated and collected by the community to date. He emphasized the need to validate resulting findings through the pursuit of designed, in vivo functional studies (item III.a in Fig. 1). To address these issues, the Jackson Laboratories, under Dr. Liu’s direction, have been using mouse models whose genomes have been modified using CRISPR/CAS9 technology, in addition to using standard mouse genetic diversity panels, to understand the in vivo physiologic significance of complex gene-phenotype relationships. He provided an example in which a mouse model for late-onset AD was developed that could be used to determine if genes and genetic variants identified from, e.g., GWAS and population sequencing studies, do indeed influence AD pathobiology (Liu et al. 2017). He also emphasized that mice could be developed like this for any gene or set of genes. He also proposed that mice developed using specific gene editing and integration technologies could possess genes exactly the same as human genes but could be developed in mice with different genetic backgrounds (i.e., mouse strains; items I.c and II.b in Fig. 1). These mice could then be used to explore the biological consequences of various interventions and environmental manipulations, including pharmacologic manipulations targeting those genes (items III.a and V.a in Fig. 1).

Lara Mangravite presented the vision and strategy behind a set of initiatives focused on the identification of the genetic basis of diseases that is open-source and community-based being pursued by Sage BioNetworks (www.sagebionetworks.org). Dr. Magravite proposed that large aggregated initiatives of the type she and her colleagues are developing could be used to identify LAVs and disease-associated variants (items I.a and I.b in Fig. 1). Dr. Mangravite provided examples of specific initiatives, including the “AMP-AD” project focusing on an integrated, systems biology approach to identifying pathways and potential therapeutic targets implicated in AD. Integrated approaches like AMP-AD could be used to identify novel IPs and potential drug targets (items III.a and III.b in Fig. 1). Dr. Mangravite also discussed some of the challenges associated with such initiatives, including data sharing from different labs, the creation of a data portal to enable queries and data deposits, and the need for harmonizing data obtained from different labs.

**Fig. 5:**
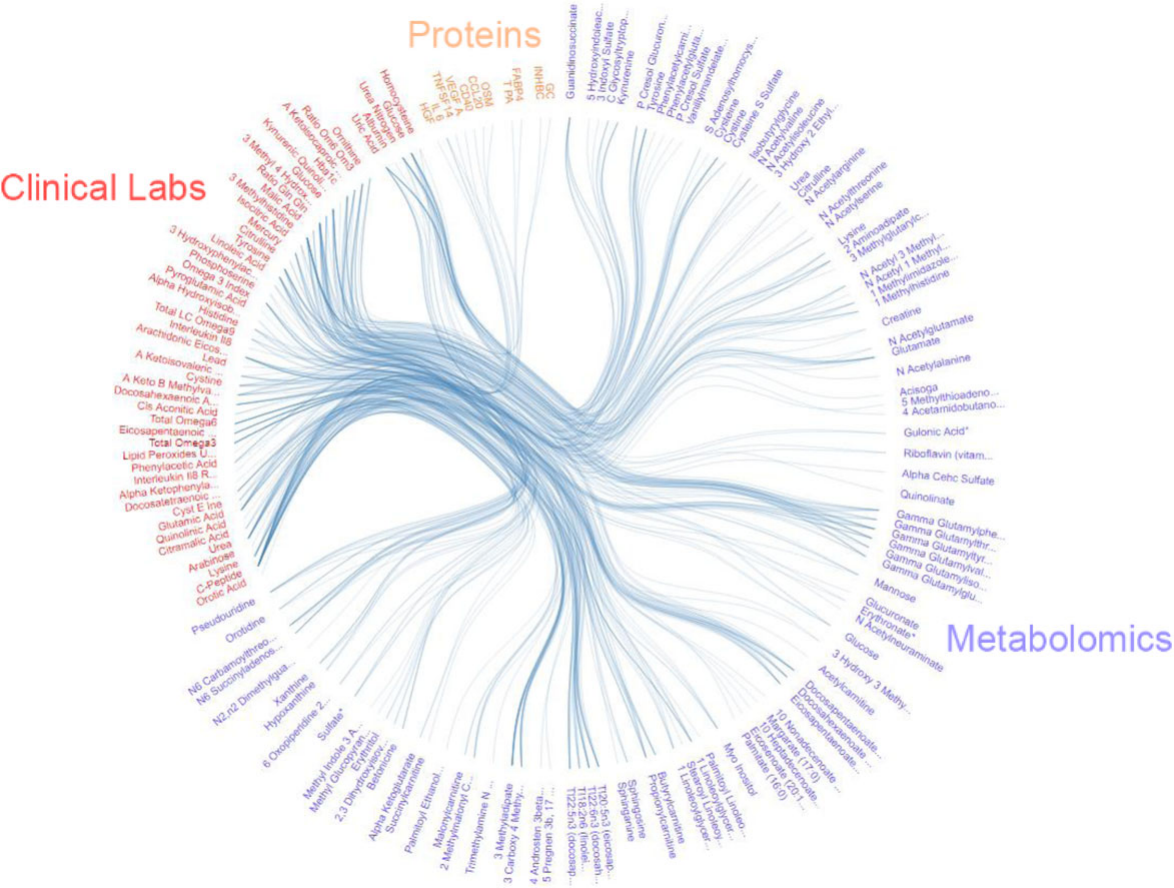
Correlations between three-different sets of aging-related analytes (proteins, metabolites and clinical lab measurements). The lines connecting the analytes suggest a definite pattern or cluster that appear to be more strongly correlated

Nathan Price discussed issues associated with the integration of genetic information, clinical data, multiple omics data, as well as environmental and lifestyle information to understand the biology of human aging. Integrated information can provide insight into the connections between genetic variants, longevity, age-related diseases, and potential IPs (items I.a, I.b, II.a, II.b, and III.a and III.b in Fig. 1). Dr. Price then discussed the creation of cohorts of individuals who could be studied longitudinally with respect to these diverse data types, and hence potentially address the role of very specific factors in human aging and longevity. He further described an initiative being pursued between the private company Arivale and the Institute for Systems Biology to collect “personal, dense, dynamic data clouds” involving different kinds of measures. In one specific substudy, the “Pioneer 100” project, 150 different clinical lab tests, ~ 1000 metabolites, ~ 400 proteins, whole-genome sequencing data, and gut microbiome data were analyzed and a number of correlations were observed that could be explored further to identify drug targets **(**Fig. 5**).**

## Further suggestions for improving translation of LAVS into drug targets

As noted, the workshop was designed to include presentations by researchers whose labs are either researching the genetic basis of human longevity, exploring genetically mediated drug targets, identifying and validating drug targets via in vitro or model organism studies, or developing resources to enable such research. The researchers were all asked to consider the question as to how the genes identified as associated with longevity might be reinforced or evaluated as drug targets and ultimately shed light on genetically mediated factors might be modulated pharmacologically to enhance or promote healthy aging. A number of points of synergy among the researchers and their scientific endeavors were obvious from the meeting. However, a number of issues were exposed where greater effort, refinement, consolidation of current efforts, and/or resource creation could enhance and catalyze this synergy. For example, purely computing-intensive data analysis (“in silico”) assessments of the relationships between genes and longevity using archived and even harmonized data were felt to be a good first step, but also severely limited, as the consensus of the participants was that there is no substitute for experimentation and empirical assessment of a genetic effect on a phenotype or as a drug target. Unfortunately, empirical studies validating drug targets can be costly and time-consuming. Therefore, either developing unique resources and/or bringing existing resources together that could motivate and prioritize efficient drug target identification studies and/or later pre-clinical development studies are crucial. As Fig. 1 indicates, this integrated and translational, cross-disciplinary activity has its roots in the flow of information starting from the design of a study to identify LAVs to the validation of a drug target. In this light, a number of suggestions were put forth as critical for advancing this holistic and integrated, yet efficient, “interventions-as-products” effort and included the following (in ascending order of costs and logistical complexity of implementation):

1. The development of better repositories for information about (1) LAVs and the genes and/or regulatory elements they reside in and affect, (2) the results of tests of causality implicating an important IP (say as the result of an MR test) for a LAV or set of LAVs, (3) the influence of an orthologous gene on longevity in different species (items I.c and II.b in Fig. 1), (4) the potential drugability of the gene or regulatory element harboring a LAV (item IV.b in Fig. 1), and (5) a list of drugs that currently target the gene or regulatory element harboring a LAV (item IV.b in Fig. 1). The current Longevity Genomics website could act as a beta-test site for such a repository of information (https://www.longevitygenomics.org). The website could also include information about, e.g., drugs currently being tested for an effect on longevity (especially since the gene targets of those drugs could be explored in human association studies; item V.b in Fig. 1), such as (1) those associated with the NIA-funded intervention testing program described by Dr. Miller (https://www.nia.nih.gov/research/dab/interventions-testing-program-itp), (2) those arising from the results of the use of resources for conducting purely computational mediation tests such as MR-Base described by Dr. Hemani (http://www.mrbase.org), and (3) those rooted in analyses involving IP-resources such as GTex described by Mr. Natoli (https://www.gtexportal.org/home/).
2. The development of better methods to identify and qualify gene orthologies across different species studied for longevity (Yanai et al. 2017). This includes, importantly, methods that consider differences in the genetic networks or pathways harboring a gene whose ortholog counterparts in other species are being tested for association with longevity (items I.c and III.b in Fig. 1). Since it is known that genes and their regulatory elements do not work in isolation, evolutionary changes that affect orthology, synteny, and other genes upstream and downstream of a target gene, are as important to consider as the target gene itself.
3. The development of novel data analysis and bioinformatics methods and tools for the integrated analysis of multi-omics data that incorporate genetic variants, transcript abundances, protein abundances, metabolite levels, etc. in order to draw more comprehensive inferences about the effects of particular factors on longevity. Such activity could involve, for example, the development of more powerful MR testing strategies, or more powerful methods for identifying and characterizing expression or protein quantitative trait loci (eQTL and pQTL; item III.b in Fig. 1).
4. The development of better assays for measuring phenotypes, both molecular and clinical, in humans that reflect the effects of the pharmacological modulation of a gene. These assays could be used to determine whether a drug that seeks to modulate the factors (i.e., IPs) thought to be influenced by LAVs are actually having an effect (items III.a and III.b in Fig. 1).
5. The development of improved “chemoinformatics” techniques and resources (i.e., information and analyses about drugs, their targets, their similarities, their amenability to modulation, etc.) for evaluating the potential of drugs and compounds to modulate an IP of interest. Chemoinformatics was not a discipline represented well at the workshop but was recognized as essential (items IV.a and IV.b in Fig. 1). In addition, knowing which gene-specific drugs target could lead to very relevant annotations for use in GWAS (items II.a and II.b), as well as motivate focused genetic association studies of genes thought to be targeted by drugs that may impact longevity (e.g., metformin or rapamycin; item V.b in Fig. 1).
6. The creation and funding of laboratory and core services that provide resources for pursuing well-controlled and designed in vitro and/or animal model functional verification studies of factors, like LAVs, identified from genetic studies (e.g., as in cell line-based transcriptomic studies of specific LAVs (Tewhey et al. 2018), CRISPR-based studies on genetic variants (Guo et al. 2017), high-throughput drug screening against cells with and without specific genetic variations (Macarron and Hertzberg 2011) studies of organoids derived from carriers of an LAV (Osaki et al. 2018) etc.; items III.a and III.b in Fig. 1).
7. The generation of longitudinal multi-omics data and profiles (e.g., genomics, transcriptomics, proteomics, metabolomics) from a large set of well-characterized age-appropriate individuals and, to the degree possible, from multiple tissues for use in characterizing the human in vivo functional consequences of LAVs (items II.b and III.b in Fig. 1; see also recent examples such as (Sebastiani et al. 2017c)). The results of such profiling could also lead to the identification of biomarkers of the aging process for use in clinical intervention studies (Justice et al. 2018).

The workshop participants recognized that greater attention will inevitably be given to the integration, reconciliation, and dissemination of the data and results of different studies of genetically mediated factors that influence longevity. This will occur because of the sheer volume of data being generated and a consequent need for determining if the results of very isolated, assay-centric, or assay-specific studies can be “triangulated” to reinforce their findings (Munafo and Davey Smith 2018). Ultimately, the workshop participants all felt that efforts focused on integration and triangulation are essential if LAVs are to be translated into drug targets. As a result, funding for collaborative projects, the construction of resources for data and study result dissemination, and better assays will be required.
